# Age‐specific reference intervals for plasma amino acids and their associations with nutrient intake in the Chinese pediatric population

**DOI:** 10.1002/imt2.70051

**Published:** 2025-05-29

**Authors:** Yang Wen, Qing Liu, Hongbo Zeng, Lina Lyu, Xuezhen He, Xin Zhang, Wentao Lyu, Weijun Chen, Yingping Xiao

**Affiliations:** ^1^ State Key Laboratory for Quality and Safety of Agro‐Products, Institute of Agro‐Product Safety and Nutrition Zhejiang Academy of Agricultural Sciences Hangzhou China; ^2^ College of Biotechnology and Bioengineering Zhejiang University of Technology Hangzhou China; ^3^ Department of Child Health Care, Children's Hospital Zhejiang University School of Medicine, National Clinical Research Center for Child Health Hangzhou China; ^4^ Children's Health Center, Beiyuan Community Service Center Yiwu China

## Abstract

A total of 2901 healthy Chinese children aged 0–12 years were enrolled in the reference group; 102 confirmed Phenylketonuria cases were included as validation individuals. Establishing the age‐specific reference intervals of 42 plasma amino acids in Chinese pediatric populations. Profiling dynamic interactions between multiple nutrient intake and amino acid change patterns.

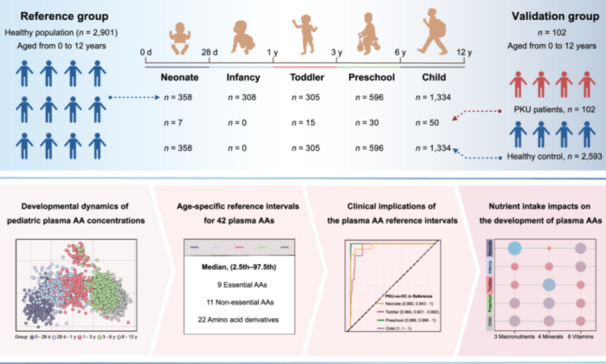

Amino acids (AAs), as fundamental building blocks of proteins and critical regulators of metabolic pathways, play indispensable roles in pediatric growth and development. Establishing age‐specific reference intervals for plasma AAs is crucial for clinical diagnostics and treatment, as physiological concentrations vary significantly across childhood due to rapid changes in development, dietary types, metabolic demands, and hormonal dynamics [1, 2]. However, existing reference ranges for pediatric populations often lack age stratification, particularly in non‐Western cohorts, leading to potential misinterpretation of AA profiles in conditions such as inborn errors of metabolism or malnutrition.

Aminoacidopathies, such as phenylketonuria (PKU) and maple syrup urine disease, are metabolic disorders caused by defects in AA metabolism [3]. These conditions are often asymptomatic in early infancy but progressively manifest with age, leading to severe outcomes including developmental delay, intellectual disability, and mortality [4]. Fortunately, early screening and intervention can significantly improve prognosis [5]. Blood AA levels have emerged as key biomarkers for disease risk prediction and therapeutic monitoring, supported by studies in aminoacidopathies, diabetes, and cancer [6, 7]. Advanced chromatographic techniques now enable precise quantification of AAs, highlighting their potential in clinical diagnostics and personalized medicine.

The association between nutrient intake and AA homeostasis remains underexplored in children. Macronutrients (proteins, fat, and carbohydrates) directly influence AA availability through dietary supply and gluconeogenic regulation [8]. Concurrently, micronutrients such as zinc (a cofactor for AA‐metabolizing enzymes) and B vitamins (critical for one‐carbon metabolism) may modulate AA conversion and utilization [9]. Emerging evidence suggests that regional dietary patterns significantly affect AA profiles [10, 11], yet comprehensive analyses integrating nutrient intake with AA variations across developmental stages are conspicuously absent from current literature.

This study addresses these gaps through two synergistic objectives: First, we analyzed the age‐specific change patterns of 42 plasma AAs, and established corresponding reference intervals from a representative cohort of 2,901 Chinese healthy children aged 0–12 years. Second, we investigate the correlation between nutrient (macronutrient, mineral, and vitamin) intakes and age‐dependent AA concentration changes. Detailed methods were provided in the Supplementary Materials and Methods. Our findings provide a more comprehensive analysis for interpreting pediatric AA profiles, with direct implications for personalized dietary interventions and metabolic disorder screening.

## RESULTS AND DISCUSSION

### Developmental dynamics of pediatric plasma AA concentrations

**Figure 1:**
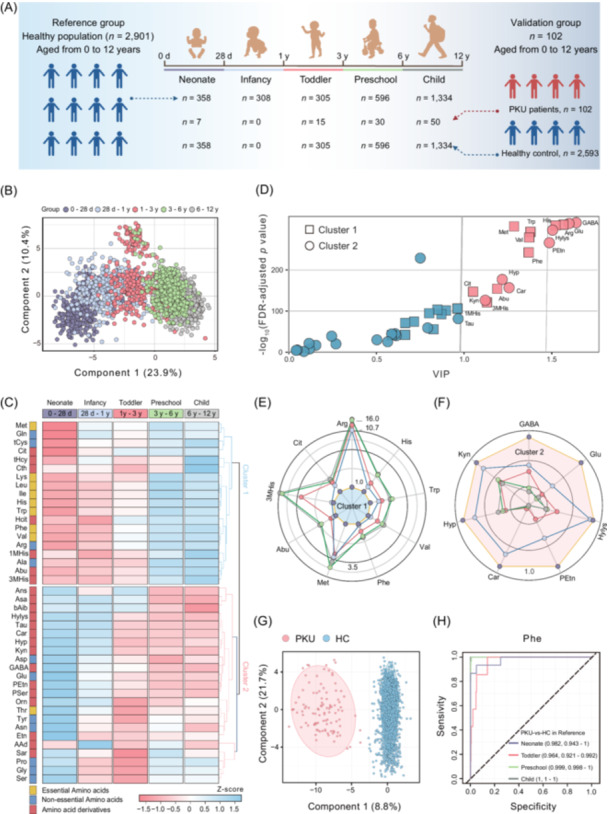
Profiles and clinical validation of age‐specific changes in 42 plasma amino acid concentrations across 0–12 years. (A) The graphic abstract of this study. (B) Orthogonal partial least squares discriminant analysis (OPLS‐DA) score plot showing the dissimilarities of plasma amino acids (AAs) concentration among different age groups (Permutation testing: *R* ^2^ *Y* = 0.923, *Q* ^2^ = 0.871). (C) Heatmap showing age‐specific changes in 42 plasma AAs, hierarchical clustering analysis based on Euclidean distance is applied to identify distinct clusters; values of the heatmap are normalized by the Z‐score by each row. (D) Identification of age‐specific differential AAs using false discovery rate (FDR)‐adjusted *p*‐value (FDR‐adjusted *p*‐value < 0.05), and Variable Importance in the Projection (VIP) scores (VIP > 1.0); Red represents VIP > 1.0, Blue represents VIP < 1.0. Radar chart showing the patterns of age‐specific change in identified AA markers from Cluster 1 (E) and Cluster 2 (F); values are fold‐changes of the normalized values in the neonate group. Clinical validation: (G) OPLS‐DA score plots show the dissimilarities of plasma AA concentrations between the PKU group and HC group. (H) Receiver operating characteristic (ROC) curves of Phenylalanine for the PKU group versus the HC group in different age groups. HC group from the reference set: *n* = 2,901 (Neonate, *n* = 358; Infancy, 308; *n *= Toddler, *n* = 305; Preschool, *n* = 596; Child, *n* = 1,334). PKU group from the validation set: *n* = 102 (Neonate, *n* = 7; Toddler, *n* = 15; Preschool, *n* = 30; Child, *n* = 50); HC, healthy control; PKU, phenylketonuria.

Developmental trajectories of 42 AAs across the defined age groups were shown to substantiate the rationality of our age division in characterizing plasma AA dynamics (Figures S1 and S2). These trajectories, when analyzed synergistically with orthogonal partial least squares discriminant analysis (OPLS‐DA) models demonstrating structural discrimination of plasma AA profiles, provide robust validation of our age‐group partitioning strategy. Then we investigated longitudinal developmental patterns in AA concentrations. The validity of these reference intervals was further evaluated through two approaches: (1) a comparative analysis against existing published reference ranges, and (2) application to a validation cohort comprising confirmed PKU patients to assess diagnostic concordance. The sample composition for each age group in the reference group and the validation group is shown in Figure 1A.

The OPLS‐DA score plot showed a clear discrimination among neonate (0–28 d), infancy (28 d–1 y), toddler (1 y–3 y), preschool (3 y–6 y), and child (6 y–12 y) (Permutation testing: *R* ^2^ *Y* = 0.923, *Q* ^2^ = 0.871, Figure 1B). Interestingly, this analytical framework identified a striking age‐dependent trend of the most plasma AA concentrations, characterized by two distinct phases: (1) a dynamic remodeling period (0–6 years) marked by significant AA changes, and (2) a metabolic stabilization phase (6–12 years) exhibiting minimal concentration variations. Additionally, segmented regression analysis further demonstrated that 5.348–6.435 years (round number = 6) was the best predicted changepoint among 42 AAs, then 78% of measured AAs exhibited ≥ two fold steeper slope magnitudes in the first developmental phase (Table S1). These change patterns likely reflect critical transitions in dietary diversification, organ development, and endocrine regulation during early childhood, necessitating stringent age‐specific reference intervals for pediatric metabolic assessments [11]. In addition, contrary to our initial hypothesis, only a few sex‐related differences in AAs were observed (Figures S2, S3 and Table S2). Sex hormones play pivotal roles in modulating AA metabolism through the regulation of transporters and catabolic enzymes. The attenuated gender differences observed in prepubertal children (0–12 years) align with low‐amplitude sex hormone fluctuations during this developmental window. However, emerging AA divergences in late childhood (6–12 years), likely foreshadow impending pubertal transitions where hormonal surges amplify metabolic dimorphism [12]. This null finding suggests that age, rather than gender, dominates AA variances in prepubertal populations.

Hierarchical clustering analysis revealed two distinct change patterns of 42 AAs (Figure 1C). OPLS‐DA modeling further identified 16 differential AAs across different age groups (Variable Importance in the Projection (VIP) > 1.0, false discovery rate (FDR)‐adjusted *p* < 0.05, Figure 1D and Table S3). These two clusters clearly exhibit complementary functions to meet the primary demands of children's growth, development, and cognitive maturation. Cluster 1 increased with age, and Cluster 2 was the opposite. Cluster 1, mainly enriched with essential AAs (histidine, tryptophan, valine, phenylalanine, methionine), exhibited progressive concentration increases with age (Figure 1E). These trends align with growth and brain development [8], as evidenced by strong correlations with anthropometric measures (Figure S4). Conversely, Cluster 2, comprising non‐essential AA (glutamate) and derivatives (γ‐aminobutyric acid, hydroxyproline), demonstrated inverse age‐related declines (Figure 1F). Notably, the age‐related decline in γ‐aminobutyric acid concentration not only balances glutamatergic excitability to protect developing neural circuits but also plays a critical role in modulating growth hormone secretion, a key driver of somatic growth [13, 14]. In summary, the 0–3 years window, marked by dramatic AAs remodeling, represents a critical period for interventions targeting lifelong neurological and physical health, making age marker AAs essential for follow‐up health examinations.

### Age‐specific reference intervals for plasma AAs of the pediatric population aged 0–12 years

Reference intervals were determined nonparametrically and correspond to the 2.5th–97.5th percentiles of the distribution (Table S4). To verify the scope of application of the present reference set, we performed a comparison of AA profiles among present RIs (China, *n* = 2,901), the Mayo Clinic (U.S.A., *n* = 877), and Thai (Thailand, *n* = 277) (Table S5). The present limits differed slightly from the global values calculated in other studies. Compared with those of the Mayo Clinic, most non‐essential AAs (Arginine, Cysteine, Glutamate, Glutamine, and Aspartate) in the 0–2 years group showed significant differences. The lower or upper limits of Taurine, Phosphoethanolamine, and Ornithine were much higher than those of the Mayo Clinic. However, these discrepancies in Arginine and Glutamine diminished in the 2–14 years group. Compared to the Thai population, the medians of the AAs were similar, and the reference intervals observed were wider, which might stem from greater variability in nutritional status across China [15, 16]. These findings challenge the universal application of Western‐derived RIs in Asian populations and advocate for locally validated intervals to avoid misdiagnosis of inborn errors of metabolism (e.g., phenylketonuria, hyperargininemia, or glutamine synthetase deficiency).

### The clinical implications of the established plasma AA reference intervals

This study validated the clinical utility of established AA reference intervals by applying them to PKU patients. PKU is a typical aminoacidopathy with the highest birth prevalence in the populations of China, Korea, and other developed countries [2]. OPLS‐DA modeling revealed a clear separation between PKU and healthy control groups (Figure 1G), with phenylalanine (Phe) identified as the most significant discriminator (VIP > 2.0, Figure S5A). In PKU patients, median Phe concentrations exceeded the reference range by >100% across all age groups (neonate, toddler, preschool, and child; Table S6), confirming its diagnostic reliability. Receiver operating characteristic curves further demonstrated high accuracy of Phe in predicting PKU (area under curve (AUC) > 0.90, Figure 1H and Figure S5B).

To enhance diagnostic specificity, we proposed a biomarker combination of Phe and tyrosine (Tyr). The Phe/Tyr model achieved superior AUC values, leveraging the metabolic relationship between these AAs (Figure S5C). Meanwhile, in samples near the PKU diagnostic threshold (*n* = 32, Figure S5D), Phe alone resulted in a 43.75% false‐negative rate (14/32 PKU cases missed); Phe/Tyr combination resulted in a 6.25% false‐negative rate (2/32 PKU cases missed). This improvement is critical for early‐stage PKU detection. Classical PKU was caused by the *PAH* gene mutants, which blocked tyrosine conversion pathways, and elevated Phe levels. Impaired phenylalanine hydroxylase (PAH) activity led to Phe accumulation and reduced Tyr synthesis [17]. The Phe/Tyr ratio thus reflected both substrate buildup and product deficiency, improving diagnostic accuracy. These findings highlight the importance of age‐specific reference intervals in diagnosing metabolic disorders. While Phe remains the gold standard for PKU diagnosis, incorporating Tyr into the diagnostic framework improves specificity and accuracy.

### Nutrient intake impacts on the development of plasma AAs

The age‐dependent divergence in AA concentrations—most differences in the 0–3 years group—aligns with rapid developmental transitions in dietary shifts (e.g., weaning). Therefore, we conducted a comprehensive association analysis (based on multivariate PERMANOVA analysis, correlation analysis, and multiple regression modeling) between integrating nutrient intake data and the developmental profiles of plasma AA concentrations in the pediatric population.

**Figure 2:**
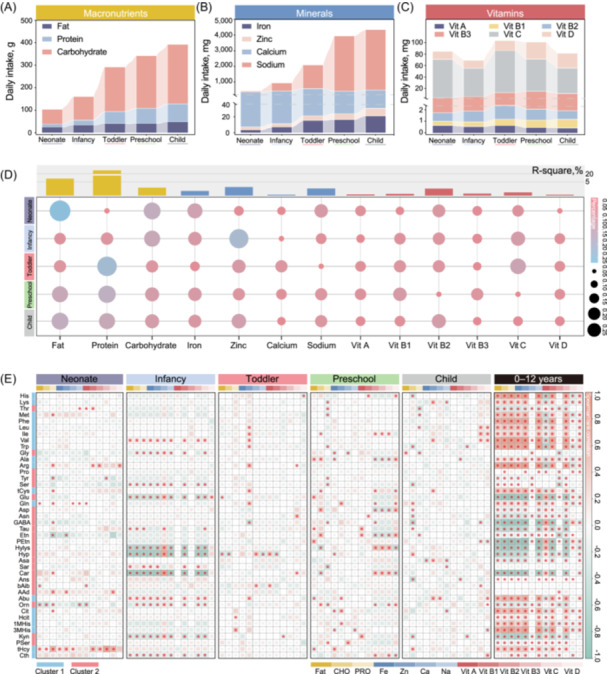
Correlation of the nutrient intake and age‐specific change patterns of plasma amino acids in the pediatric population. Daily intake of nutrients including macronutrients (A), minerals (B), and vitamins (C) in children aged 0–12 years, integrated various studies across different age groups. (D) PERMANOVA analysis of the nutrients affecting the plasma amino acids (AAs) concentrations using multivariate models; bubble chart showing the explained variation represented by R‐squared in different age groups; histogram showing the explained variation of nutrients across the pediatric population aged 0 to 12 years. (E) Spearman's rank correlations between the daily intake of nutrients and the concentrations of 42 plasma AAs in the pediatric population across 0–12 years; red star represents significance, *p*‐value < 0.05; the color represents Spearman's rank correlations. *n* = 2,901 (Neonate, *n* = 358; Infancy, 308; *n *= Toddler, *n* = 305; Preschool, *n* = 596; Child, *n* = 1,334). Macronutrients including fat, protein (PRO), and carbohydrate (CHO); minerals including iron (Fe), zinc (Zn), calcium (Ca), and sodium (Na); vitamins including vitamin A, B1, B2, B3, C, and D.

Macronutrients and micronutrients exhibited distinct age‐dependent intake trajectories in children aged 0–12 years (Figure 2A–C, Figure S6, and Table S7). Protein, carbohydrate, zinc, and iron intake displayed a continuous upward trend from neonate, aligning with escalating growth demands. In contrast, fat and vitamin intake remained stable across age groups. Multivariate PERMANOVA analysis revealed that nutrients explained variation of plasma AA profiles shifted dramatically with age (Figure 2D). During the 0 to 1 y critical window, fat, carbohydrates, and zinc dominated AA variability, likely due to their roles in energy provision and enzyme cofactor activity [1, 6]. By contrast, protein emerged as the primary driver after age 2, reflecting its direct contribution to AA synthesis for tissue growth. In addition, calcium and vitamins B1/B3/D showed minimal explained variation. However, the possibility that vitamin D may affect AA metabolism through systemic indirect regulatory effects cannot be ruled out. For example, the synergistic effect of calcium and vitamin D might influence the AA profiles by modulating skeletal muscle metabolism [18].

Spearman's correlation analysis and multiple regression modeling demonstrated that nutrient intake exhibited a positive correlation with Cluster 1 and a negative correlation with Cluster 2, suggesting a strong relationship between nutrient intake and AA development (Figure 2E and Table S8). Longitudinal analysis further highlighted that macronutrients, zinc, and vitamin B2 played a direct role in shaping the AA change patterns. Notably, despite low protein intake during the neonate and infancy, it showed negative correlations trends with key AAs such as glutamate/glutamine (Standardized regression coefficient: −4.376/−0.108, *p* = 0.086), GABA (Standardized regression coefficient: −3.723, *p* = 0.088), and arginine (Standardized regression coefficient: −0.958, *p* = 0.022, Table S9), suggesting its precursor role in neurodevelopment. These findings align with previous studies emphasizing the critical role of protein and zinc in early growth and neurodevelopment, particularly during the first 3 years of life [19, 20]. This study revealed that multiple AAs exhibited consistent trends in their associations with nearly all nutrients during specific age periods, highlighting the complex interplay between nutrient intake and AA metabolism throughout childhood development. We acknowledge that while our study quantifies the contributions of nutrient intake to plasma AA concentrations, the mechanistic origins of these variations, whether stemming from de novo synthesis or utilization efficiency, remain unresolved. Especially, early gut immaturity may synergize nutrient bioavailability, microbial metabolism, and enterohepatic development to shape plasma AA profiles [20]. Future studies should explore age‐specific nutrient‐AAs relationships within dynamic gut ecosystem maturation frameworks.

## CONCLUSION

In summary, this study established a more comprehensive reference interval of Chinese pediatric plasma AAs and elucidated the associations between the nutrients intake and amino acid metabolic trajectories. These findings provide useful information to assess the diagnostic value of AA profiles in metabolic diseases and design the targeted nutritional interventions tailored to pediatric growth stages.

## METHODS

Detailed methods and procedures were provided in the Supporting Information.

## AUTHOR CONTRIBUTIONS

**Yang Wen**: Conceptualization; investigation; funding acquisition; writing—original draft; methodology; validation; visualization; writing—review and editing; software; formal analysis; data curation. **Qing Liu**: Data curation; formal analysis; visualization; methodology; writing—review and editing. **Hongbo Zeng**: Data curation; formal analysis. **Lina Lyu**: Formal analysis; data curation. **Xuezhen He**: Supervision. **Xin Zhang**: Funding acquisition. **Wentao Lyu**: Conceptualization; writing—review and editing; formal analysis; project administration; supervision. **Weijun Chen**: Conceptualization; investigation; writing—review and editing; project administration; resources; supervision. **Yingping Xiao**: Funding acquisition; conceptualization; investigation; writing—review and editing; methodology; project administration; resources; supervision.

## CONFLICT OF INTEREST STATEMENT

The authors declare no conflicts of interest.

## ETHICS STATEMENT

All experimental procedures were approved by the Children's Hospital affiliated with Zhejiang University School of Medicine Ethics Committee (Approval Nos. 2022‐IRB‐253). All healthy subjects and patients included in the study signed informed consent forms and agreed to participate in this study, as well as to the publication of related content.

## Supporting information


**Figure S1.** Scatterplot of 42 plasma amino acid concentrations by age, split by gender.
**Figure S2.** Trends of the age‐specific distribution of each amino acid concentration.
**Figure S3.** Comparison of plasma amino acid concentrations between sexes in different age groups, related to Table S2.
**Figure S4.** Correlation of plasma amino acids with age, weight, and height.
**Figure S5.** Identification of differential amino acids in plasma between phenylketonuria patients and healthy control, related to Figure 1 and Table S7.
**Figure S6.** Proportions of daily nutrient intake including macronutrients (A), minerals (B), and vitamins (C) in children aged 0 to 12 years.

**Table S1.** Segmented regression analysis of age‐specific plasma amino acid concentrations across 0–12 years, related to Figure S1.
**Table S2.** Differences in plasma amino acid concentrations between sexes, related to Figure S2 and S3.
**Table S3.** Comparison of 42 plasma amino acid concentrations from the pediatric population among different age groups, related to Figure 1.
**Table S4.** Age‐specific reference intervals for the 42 plasma amino acids of the pediatric population aged 0–12 years.
**Table S5.** Comparison of plasma amino acid reference intervals between the present reference set (China) and Mayo Clinic (U.S.A.), Thai (Thailand).
**Table S6.** Comparison of plasma amino acid concentrations between phenylketonuria patients and healthy control, related to Figure 1 and Figure S3.
**Table S7.** Summary of daily nutrients intake for pediatric population aged 0 to 12 years from multiple studies, related to Figure 2.
**Table S8.** Spearman's correlations between the daily intake of nutrients and the concentrations of 42 plasma amino acids in the pediatric population, related to Figure 2.
**Table S9.** Multiple regression modeling of multiple nutrients intake and the plasma amino acid concentrations.
**Table S10.** Demographics of the entire reference group.

## Data Availability

The characteristics and sources of the used data set were shown in Supporting Information: Tables S1–S10. Supplementary materials (methods, figure, table, graphical abstract, slides, videos, and Chinese translated version) may be found in the online DOI or iMeta Science http://www.imeta.science/.
