# Supplementary material for: Age‐specific reference intervals for plasma amino acids and their associations with nutrient intake in the Chinese pediatric population
Source: Imeta. 2025 May 29;4(3):e70051. doi: 10.1002/imt2.70051 (PMC12130562; doi:10.1002/imt2.70051)
Supplement: Supplementary file 1 — Figure S1. Scatterplot of 42 plasma amino acid concentrations by age, split by gender. Figure S2. Trends of the age‐specific distribution of each amino acid concentration. Figure S3. Comparison of plasma amino acid concentrations between sexes in different age groups, related to Table S2. Figure S4. Correlation of plasma amino acids with age, weight, and height. Figure S5. Identification of differential amino acids in plasma between phenylketonuria patients and healthy control, related to Figure 1 and Table S7. Figure S6. Proportions of daily nutrient intake including macronutrients (A), minerals (B), and vitamins (C) in children aged 0 to 12 years. [file IMT2-4-e70051-s001.docx]

**Support information for**

**Age-****Specific Reference Intervals for Plasma Amino Acids and Their Associations with Nutrient Intake in the Chinese Pediatric Population**

**Running title:** Age-Specific Plasma Amino Acid Reference Intervals in Chinese Children

Yang Wen^1#^, Qing Liu^2#^, Hongbo Zeng^1^, Lina Lyu^3^, Xuezhen He^4^, Xin Zhang^3^, Wentao Lyu^1*^, Weijun Chen^3*^, Yingping Xiao^1*^

^1^State Key Laboratory for the Quality and Safety of Agro-Products, Institute of Agro-Product Safety and Nutrition, Zhejiang Academy of Agricultural Sciences, Hangzhou, 310021, China

^2^College of Biotechnology and Bioengineering, Zhejiang University of Technology, Hangzhou, 310032, China

^3^Department of Child Health Care, Children's Hospital, Zhejiang University School of Medicine, National Clinical Research Center for Child Health, Hangzhou, 310052, China

^4^Children's Health Center, Beiyuan community service center, 322000, Yiwu, China

^#^These authors contributed equally: Yang Wen, Qing Liu

^*^Correspondence: [xiaoyp@zaas.ac.cn](mailto:xiaoyp@zaas.ac.cn) (Yingping Xiao); [chenweijun@zju.edu.cn](http://chenweijun@zju.edu.cn) (Weijun Chen); [lvwt@zaas.ac.cn](mailto:lvwt@zaas.ac.cn) (Wentao Lyu).

**MATERIALS AND METHODS**

**Study design and population**

We conducted a cross-sectional study about profiling plasma amino acid (AA) levels of a healthy pediatric population in China aged 0–12 years. This study was approved by the Children's Hospital affiliated with Zhejiang University School of Medicine Ethics Committee, and obtained from the parents or participants of informed consent. **Figure 1A** depicts the basic characteristics of participants.

To establish accurate and clinically relevant reference intervals for 42 plasma AAs in Chinese children across 0–12 years, we first referred to the age staging of children in the 10th edition of Pediatrics, a textbook of clinical medicine for Chinese institutions of higher learning, which reflect clinically meaningful transitions in pediatric development. That was mainly determined based on three key physiological transitions: (1) developmental milestones (rapid organogenesis particularly brain, liver and gut maturation, and evolution of intestinal microbiome), (2) dietary transitions (breastmilk to complementary foods, then to family diet), and (3) metabolic maturation (liver enzyme development and protein utilization efficiency).

The reference group contained 2,901 normal children aged from 0 to 12 years, and is divided into five age brackets: Neonate (0–28 d), n = 358; Infancy (28 d–1 y), *n* = 308; Toddler (1 y–3 y), *n* = 305; Preschool (3 y–6 y), *n* = 596; Child (6 y–12 y), *n* = 1,334. All participants recorded the information on age and gender, and a partial of them (*n* = 1,599, 55.1%) additionally recorded the information on weight, height, and BMI. Detailed participants’ characteristics are presented in **Table S10**.

Blood samples were collected from participants of health examinations in China. The inclusion criteria of the study population followed the Clinical and Laboratory Standards Institute (CLSI) document C28‐A3 and Chinese Health Industry Standards (WS/T 402‐2012) guidelines [1,2]. We excluded participants with clinical diseases (like metabolic, renal, hepatic, cardiac, or muscular diseases), premature infants ( < 37 weeks), malnutrition, and obesity.

The validation group contained 102 confirmed Phenylketonuria (PKU) patients aged from 0 to 12 years, matched by age and gender. All participants derived from the same Chinese population as the reference cohort were enrolled from January 2022 to November 2023 at the Biosan Medical Testing Laboratory in Zhejiang, China, and we obtained permission to retrieve their values of plasma AA concentrations and characteristics. Detailed participants’ characteristics are presented in **Table S6**.

**Amino acid profiling**

In this study, all the tests were performed at the Biosan Medical Testing Laboratory in Zhejiang, China to ensure consistency in the testing process and to meet required analytical performance specifications. We profiled 42 plasma AAs (classified as 9 Essential AAs, 11 Non-essential AAs, and 22 AA derivatives) using liquid chromatography-tandem mass spectrometry (LC-MS/MS), and quantified AA concentrations based on calibration curves. Calibration curves were prepared for a serial dilution in stock pooled plasma using AA standards and their corresponding isotope-labeled reference standard.

**Sample Preparation**

Participants ( > 3 years) were required to faste 8–12 hours; Participants (1 year–3 years) fasted 4-8 hours; Participants ( < 1 year) fasted 2-3 hours post-feeding per neonatal protocols. All plasma samples were collected at 7:00 – 9:00, and were collected from fasting venous blood (2 mL) in plasma separation tubes. Only one attempt to collect blood per participant was allowed. Within 2 hours after sample collection, centrifuge at 3,000 g for 15 mins, and supernatant was collected. Plasma stored at -80°C, and would be analyzed for AA levels within one week.

Extract 50 µL of plasma sample into a 1.5 mL centrifuge tube, and add 200 µL of methanol containing internal standards and 20 mg/mL DTT (dithiothreitol) with 0.1% formic acid. Vortex the mixture thoroughly, and centrifuge at 14,000 rpm at 4°C for 10 minutes. Transfer 200 µL of the supernatant to a 2 mL centrifuge tube, and evaporate it under nitrogen gas at 40°C. Reconstitute the dried sample in 100 µL of mobile phases A and B at a 1:2.5 ratio (70% acetonitrile). After thorough vortex mixing for 3 minutes, centrifuge at 14,000 rpm at 4°C for 10 minutes, and transfer the supernatant through a 0.22-μm membrane filter to obtain the test solution, which is then transferred to an injection vial for AAs quantitative determination.

**Amino acid determination**

42 AAs analysis was conducted on API 4500 LC-MS/MS system (Triple Quad™ 4500MD, AB Sciex, MA, USA) with an ACQUITY UPLC BEH Amide Column (1.7 μm, 2.1 mm × 50 mm). The column temperature was set at 35°C, and the mobile phase consisted of solution A: 0.1% (v/v) aqueous formic acid with 20% (v/v) acetonitrile with 10 mM ammonium formate and solution B: 90% (v/v) acetonitrile solution with 0.1% (v/v) formic acid with 10 mM ammonium formate. MS analyses were carried out using electrospray ionization (ESI) and multiple reaction monitoring (MRM) scans in the positive ion mode.

**Literature integration of the nutrient intake in pediatric population**

To investigate the association between the nutrient intake and plasma AAs, PubMed and Scopus were searched for literature published about nutrient intake of Chinese children (0–12 years old) between January 2010 and January 2025. Based on a review of nutrient intake in Chinese (including 83 literatures), we updated its reference intervals (including 147 literatures) [3]. After study selection and quality assessment, a total of 13 nutrients were included in the analysis, classified as macronutrients (fat, protein, and carbohydrate), minerals (iron, zinc, calcium, and sodium), and vitamins (vitamin A, B1, B2, B3, C and D). Detailed information of data set and its sources was listed in the **Table S7**.

**Statistical analysis**

Statistical analysis was performed using the IBM SPSS Statistics version 21.0 (IBM Corp., Armonk, NY, USA) software package (SPSS Inc, Chicago, IL) and GraphPad Prism v10.0 (GraphPad Software, Inc., La Jolla, CA). All data were analyzed according to CLSI C28-A3c guidelines. Outliers were identified using the ROUT method [4]. Age and sex partitions were determined by visually inspecting the distribution and scatter plots for overall trends. Orthogonal partial least squares discrimination analysis (OPLS-DA) model was used to analyze the dissimilarities of plasma AA concentrations and provide validation of the age-group partitioning strategy. Reference intervals were determined nonparametrically and correspond to the 2.5th–97.5th percentiles of the distribution, and Sex-stratified reference intervals were established for affected analytes. Comparisons of plasma AAs among different ages were performed with the nonparametric Kruskal‐Wallis test. A two‐tailed *p* < 0.05 was considered to be statistically significant. For continuous variables, the independent samples *t*-test or two-tailed Mann–Whitney *U* test was used for comparisons between two independent groups. Segmented regression analysis was used to validate the biphasic change pattern of plasma AAs across 0–12 years.

OPLS-DA model with Variable Importance in the Projection (VIP) scores and false discovery rate (FDR)-adjusted *p* value was performed to identify the key biomarker between healthy population and PKU patients. Receiver operating characteristic (ROC) analysis was used to evaluate the diagnostic performance of AAs for PKU detection. Selected Borderline PKU cases were selected according to the reference intervals of Phe; then False-negative rate of the Phe alone and Phe/Tyr combination measurement was analyzed to expand their clinical utility.

The correlation of plasma AAs with age, weight, height, and nutrients was analyzed by Spearman’s rank correlation. Then, an integrated analysis of multivariate PERMANOVA, Spearman’s correlation analysis, and multiple regression modeling (standardized regression coefficients, 95% Cl and statistical significance values) was used to elucidate the relationships between multiple nutrient intake and specific plasma AA concentrations.

**References**

1. Horowitz, Gary L., Sousan Altaie, James C. Boyd, Ferruccio Ceriotti, Uttam Garg, Paul Horn, Amadeo Pesce, et al. 2008. “Defining, establishing, and verifying reference intervals in the clinical laboratory; approved guideline—CLSI document C28‐A3.” Thirdth edn. Clinical and Laboratory Standards Institute, Wayne, PA.
2. National Health Commission of People’s Republic of China. WS/T 402–2012 Define and Determine the Reference Intervals in Clinical Laboratory. Beijing: Standards Press of China; 2013:1‐9.
3. Ayling, Katie, Rongrong Li, Leilani Muhardi, Alida Melse-Boonstra, Ye Sun, Wei Chen, Urszula Kudla. 2023. “Systematic literature review of the nutrient status, intake, and diet quality of Chinese children across different age groups.” Nutrients 15(6):1536. <https://doi.org/10.3390/nu15061536>
4. Motulsky, Harvey J., Ronald E. Brown. 2006. “Detecting outliers when fitting data with nonlinear regression–a new method based on robust nonlinear regression and the false discovery rate.” BMC Bioinformatics 9(7):123. <https://doi.org/10.1186/1471-2105-7-123>

**Supplementary Figures**

**Figure S1**. Scatterplot of 42 plasma amino acid concentrations by age, split by gender. *n* = 2,901 (Neonate, *n* = 358; Infancy, 308; Toddler, *n* = 305; Preschool, *n* = 596; Child, *n* = 1,334).

**Figure S2**. Trends of the age-specific distribution of each amino acid concentration. *n* = 2,901 (Neonate, *n* = 358; Infancy, 308; Toddler, *n* = 305; Preschool, *n* = 596; Child, *n* = 1,334). Values represent by mean ± 95% Cl.

**
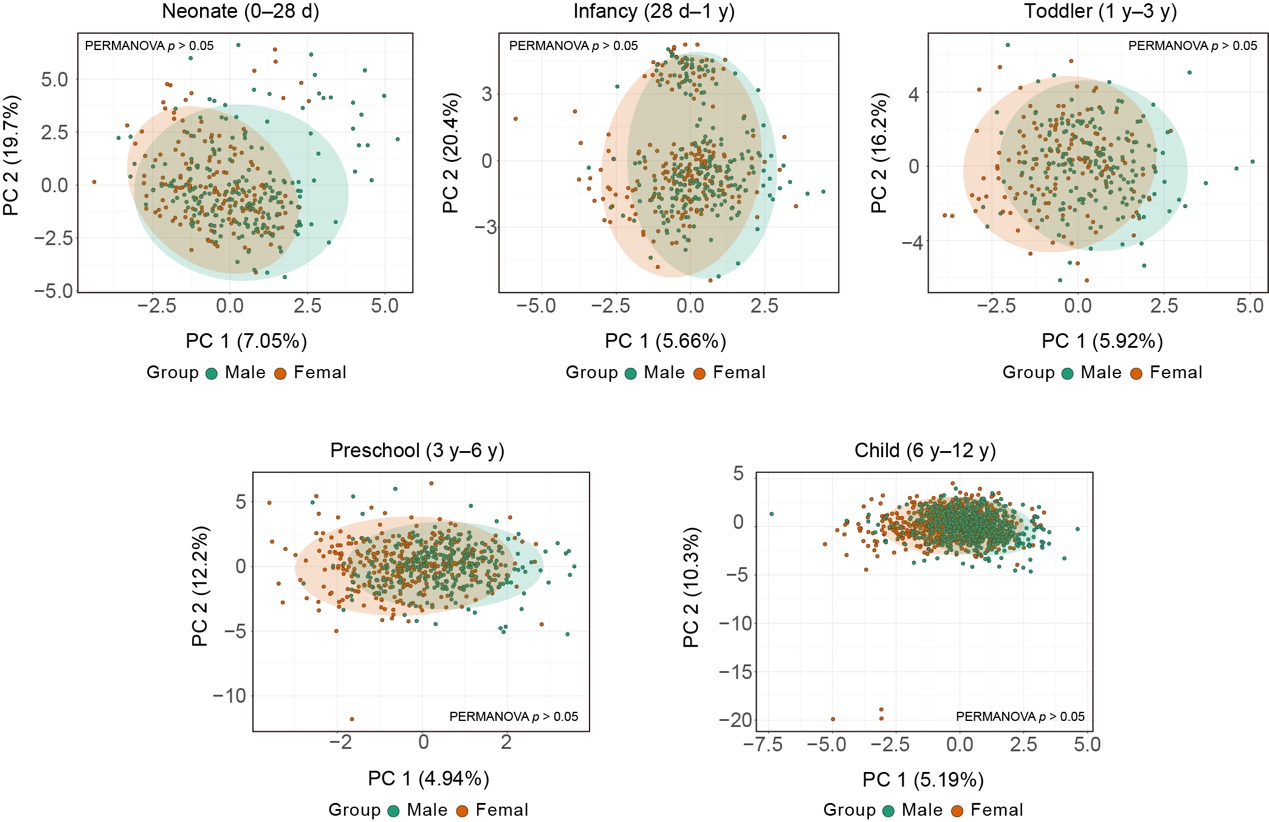
**

**Figure S3**. Comparison of plasma amino acid concentrations between sexes in different age groups, related to Table S2. Principal coordinate analysis (PCoA) was employed, using Bray-Curtis dissimilarity, to examine the dissimilarities of plasma amino acids between sexes. PERMANOVA evaluated significance (*p* < 0.05). Neonate (0–28 d), *n* = M: 190/F: 168; Infancy (28 d–1 y), *n* = M: 166/F: 142; Toddler (1 y–3 y), *n* = M: 158/F: 147; Preschool (3 y–6 y), *n* = M: 280/F: 316; Child (6 y–12 y), *n* = M: 629/F: 705.

**Figure S4**. Correlation of plasma amino acids with age, weight, and height. The heatmap shows the associations between 42 plasma amino acids and age, weight, and height, respectively. Values are shown as Spearman’s rank correlation, and “X” represents the corresponding *p*-value > 0.05.

**Figure S5.** Identification of differential amino acids in plasma between phenylketonuria patients and healthy control, related to Figure 1 and Table S6. (A) Variable importance in the projection (VIP) scores of plasma amino acids between the PKU groups and HC groups. (B) Receiver operating characteristic (ROC) curves of Phe for the PKU group *versus* the HC group. (C) ROC curves of Phe/Tyr for the PKU group versus the HC group. (D) False-negative rate of the Phe and Phe/Tyr measurement in borderline PKU cases. PKU group: *n* = 102; HC group: *n* = 2,593 (except for infancy, 28 d–1 year). PKU, phenylketonuria; HC, healthy control; Phe, phenylalanine; Tyr, tyrosine.

**Figure S6.** Proportions of daily nutrient intake including macronutrients (A), minerals (B), and vitamins (C) in children aged 0 to 12 years. Details were shown in Table S7.
